# Reactive Insertion of PEDOT-PSS in SWCNT@Silica Composites and its Electrochemical Performance

**DOI:** 10.3390/ma13051200

**Published:** 2020-03-06

**Authors:** Halima Djelad, Abdelghani Benyoucef, Emilia Morallón, Francisco Montilla

**Affiliations:** 1 Laboratoire des Sciences et Techniques de l’Eau, University of Mascara, Bp 763 Mascara 29000, Algeria; halima@ua.es (H.D.); a.benyoucel@univ-mascara.dz (A.B.); 2 Departamento de Química Física e Instituto Universitario de Materiales, Universidad de Alicante, Ap. 99, E-03080 Alicante, Spain; morallon@ua.es

**Keywords:** PEDOT-PSS, SiO_2_, sol-gel, hybrid materials, ferrocene

## Abstract

Hybrid silica-modified materials were synthesized on glassy carbon (GC) electrodes by electroassisted deposition of sol-gel precursors. Single-wall carbon nanotubes (SWCNTs) were dispersed in a silica matrix (SWCNT@SiO_2_) to enhance the electrochemical performance of an inorganic matrix. The electrochemical behavior of the composite electrodes was tested against the ferrocene redox probe. The SWCNT@SiO_2_ presents an improvement in the electrochemical performance towards ferrocene. The heterogeneous rate constant of the SWCNT@SiO_2_ can be enhanced by the insertion of poly(3,4-Ethylendioxythiophene)-poly(sodium 4-styrenesulfonate) PEDOT-PSS within the silica matrix, and this composite was synthesized successfully by reactive electrochemical polymerization of the precursor EDOT in aqueous solution. The SWCNT@SiO_2_-PEDOT-PSS composite electrodes showed a heterogeneous rate constant more than three times higher than the electrode without conducting polymer. Similarly, the electroactive area was also enhanced to more than twice the area of SWCNT@SiO_2_-modified electrodes. The morphology of the sample films was analyzed by scanning electron microscopy (SEM).

## 1. Introduction

The need for sensors in molecular analysis has stimulated the development of new electrocatalytic materials and electrochemical devices. Most of these materials can be categorized as nanomaterials (metal nanoparticles, nanotubes, graphene, etc.) exhibiting novel electronic, optical or mechanical properties [1,2,3]. Single-wall carbon nanotubes (SWCNTs) comprise an interesting group of materials with applications in electrocatalysis that can be employed as sensing elements [4,5,6,7]. Electrodes modified with carbon nanotubes have been applied to the electrochemical detection of a large number of species (dopamine, uric acid, nicotinamide adenine dinucleotide, ascorbic acid, tyrosine, insulin, etc.). These nanotubes have been also used as transducers for direct electron transfer to redox enzymes [8,9,10]. A major drawback of carbon nanotubes is their strong tendency to aggregate when they are deposited on a substrate, because of the strong π–π attractive interaction between the tube walls. The aggregation of nanotubes produces the loss of some of the physicochemical properties in the macroscopic measurement. Therefore, the dispersion of the active material onto the supporting electrode, keeping the nanoscopic character of the electrocatalytic material, is a key point for the development of sensors with superior properties. The immobilization of the nanomaterial can be performed inside a porous inorganic matrix, such as silica, in a simple way following sol-gel methodologies. This technique provides an easy method to encapsulate chemical species in a stable host. These modifiers provide enhanced electrocatalytic activity related to an improvement of the heterogeneous electron transfer rate [11]. The electrocatalytic activity of single-wall carbon nanotubes (SWCNTs) dispersed within a SiO_2_ matrix (SWCNT@SiO_2_) was examined against redox probes presented in previous works. This material improves heterogeneous rate transfer of the electrodes for all the common redox probes.

Following this approach, several silica nanocomposites functionalized with carbon materials have been employed as electrode modifiers in several applications [12]. The development of highly sensitive electrochemical sensors, e.g., biosensors, was achieved by the combination of conducting polymers with graphene or carbon nanotubes, which provide to the composite high electrical conductivity, active surface area and fast electron transfer rate [13,14,15].

However, most of SWCNTs incorporated in silica remain isolated from the underlying electrode with no direct electrical connection [16,17]. In this work, the electrochemical performance of carbon nanotubes dispersed in silica was tested against the ferrocene redox probe. This species is an outer-sphere redox probe. The electrochemical reaction happened without any adsorption step and showed low reorganization energy upon redox transitions [18]. For those reasons, ferrocene has been routinely used to investigate electron-transfer kinetics in chemically modified electrodes [19,20] since this redox probe is usually incorporated as a mediator in electrochemical biosensors [21,22,23].

The objective of the present work was to make a better electrical contact between the dispersed carbon nanotubes in silica gels by growing conductive molecular wires between the SWCNT and the supporting electrode. Due to the low solubility of the 3,4-ethylendioxythiophene (EDOT) monomer in aqueous solutions, it is necessary to add a surfactant to the solution. Poly (sodium 4-styrenesulfonate) (PSS) behaves as a surfactant but also as an electrolyte that supplies enough conductivity to the solution, remaining inserted in the polymer film as a doping agent [24]. These poly(3,4-Ethylendioxythiophene)-poly(sodium 4-styrenesulfonate) PEDOT-PSS films find applications as the transducers of biosensors for peroxides, or as mediators for redox enzymes [25,26].

We chose PEDOT-PSS since this polymer presents a poor electrocatalytic performance for the electron transfer to the ferrocene redox probe [11]. In that manner, if any electrocatalytic effect is observed for the composite material, this effect could be related only to the electrical wiring of the SWCNT with the electrode and not to the mere presence of the polymer. The morphology of the nanocomposite electrodes was characterized by electron microscopy and the electrochemical performance of the new nanocomposite electrode, SWCNT@SiO_2_-PEDOT-PSS, was tested against a model redox probe. The effect of the nanotubes within the silica layer was studied in separate electrodes to confirm the wiring effect provided by the conducting polymer.

## 2. Materials and Methods

SWCNTs were purchased from Cheap Tubes Inc. (Brattleboro, VT, USA, purity 95%, 1–2 nm diameter) and were used without further purification. 3,4-Ethylendioxythiophene (EDOT), poly (sodium 4-styrenesulfonate) (PSS), ferrocenium hexafluorophosphate (Fc), tetraethyl orthosilicate and ethanol (EtOH) were purchased from (Sigma-Aldrich, Madrid, Spain). Potassium chloride, hydrochloric acid and sulfuric acid were purchased from Merck Company and all the solutions were freshly prepared with deionized water obtained from an Elga Labwater Purelab Ultra system.

Cyclic voltammetry (CV) experiments were carried out in a conventional three-electrode cell under N_2_ atmosphere. A platinum wire was used as the counter electrode. The working electrode used was a glassy carbon (GC, geometric area = 0.07 cm^2^, Carbone Lorraine, model V-25) rod. The current density was calculated from this geometric area. The GC electrode was submitted to the following cleaning procedure before each experiment. The GC was polished with fine emery paper and subsequently rinsed with ultrapure water. Potentials were measured against the reversible hydrogen electrode (RHE) immersed in the same electrochemical cell. An EDAQ EA163 model potentiostat coupled to an EG&G Parc Model 175 was used for both the synthesis and electrochemical testing of the samples. The surface morphology of modified GC electrodes was studied by scanning electron microscopy (SEM) and images were obtained using the field emission scanning electron microscope (FESEM).

Before deposition, GC electrodes were cleaned by polishing with alumina slurries and were rinsed with water. The precursor of silica was synthesized by a mixture of 2.69 mmol of TEOS, 8.2 mL of EtOH and 5.8 mL of a solution of 0.01M HCl with 0.46 M KCl. This was stirred for 1 h in a closed vial. After 2 h, the resulting sol was submitted to evaporation by vacuum heating until the complete removal of the released ethanol from alkoxide hydrolysis was achieved.

Stable SWCNT aqueous suspensions were obtained as follows: 20 mg of SWCNT were poured into a vial containing 20 mL of 1% poly (4-styrenesulfonic acid) aqueous solution. The carbon nanotubes were dispersed and suspended by the application of an ultrasonic field by a VIRTIS probe (Virsonic 475, 475W maximum output power) at 1 min intervals for 1 h. To avoid overheating, samples were ice-cooled between sonication intervals.

For the preparation of SWCNT@SiO_2_ on GC electrodes, 2.52 mL of the SWCNT solution were poured into the silica precursor solution. This mixture containing SWCNT and the hydrolyzed silica precursor was placed in an electrochemical glass cell which contained a platinum wire counter electrode and a reversible hydrogen reference electrode to proceed with the electroassisted deposition. Further details of the deposition method are provided in other research [11,27].

EDOT electropolymerization was carried out in an aqueous medium prepared by dissolving 1.46 g PSS in 10.0 mL ultrapure water; 53 μL EDOT monomer were then added and the resulting solution was stirred in an ultrasonic bath for 30 min.

At least 3 replicas of the synthesized electrodes were obtained. The different electrodes were tested with distinct redox probes, obtaining peak separation variations of less than 6 mV between the different samples. The most representative electrodes of each species are shown in this work.

## 3. Results and Discussion

### 3.1. Electrochemical Behavior of Modified Electrodes

**Figure 1:**
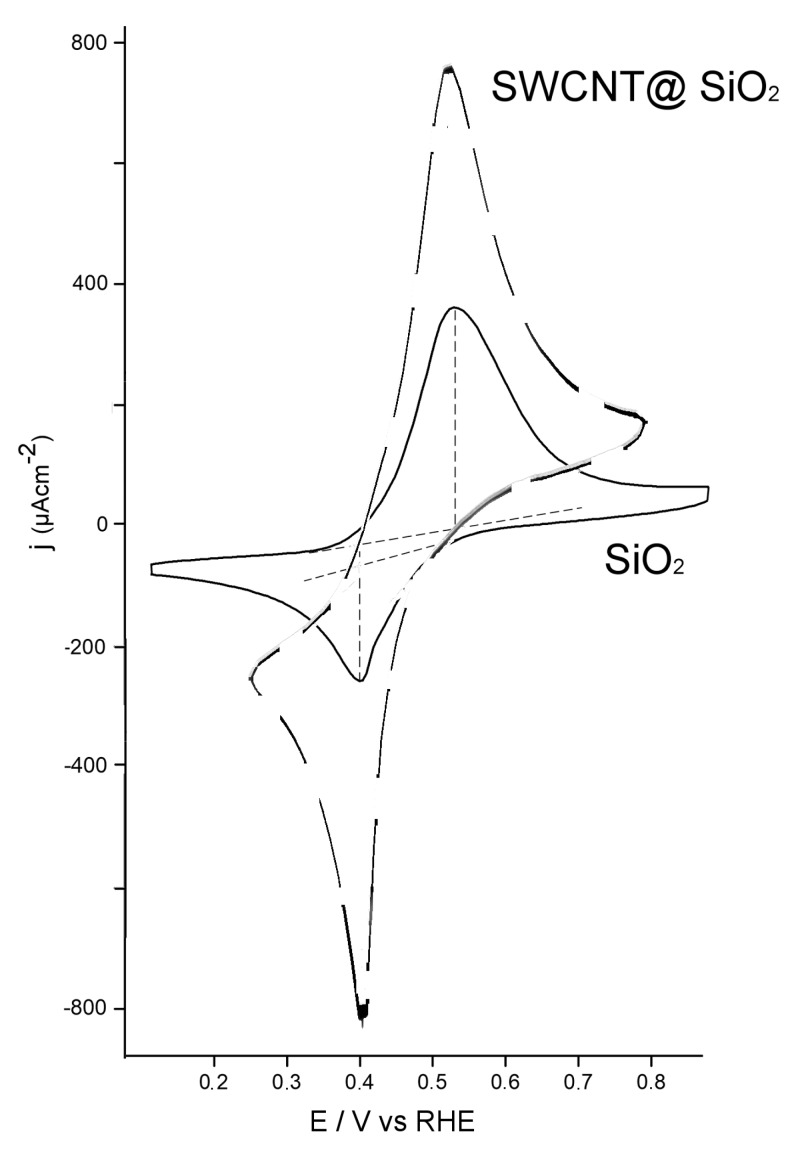
Stabilized cyclic voltammograms of a silica-modified electrode (solid line) and a Single-Wall Carbon Nanotubes in a silica matrix (SWCNT@SiO_2_)-modified electrode (dashed line) in a solution of 1.0 mM FcPF_6_ in 0.5M H_2_SO_4_. Scan rate of 100 mV s^−1^.

The redox chemistry of ferrocene was studied using cyclic voltammetric (CV) with the modified electrodes. Ferrocene/ferricenium (Fc/Fc^+^) is one of the most common outer-sphere redox probes and it is very sensitive to the active sites for electron transfer in SWCNTs as it may react through both nanotube walls and tips [7]. The test solution was prepared with 1.0 mM ferrocenium hexafluorophosphate (FcPF_6_) in a 0.5 M sulfuric acid solution. The resultant stabilized CV curves are shown in Figure 1. 

Electrodes were modified by silica films obtained by electroassisted deposition at a current density of 2.5 mA cm^−2^. For these experiments, the total charge applied to the deposition of silica Q_silica_ was 150 mC cm^−2^.

Figure 1 shows the stabilized cyclic voltammogram of a GC/SiO_2_ (150 mC·cm^−2^) electrode immersed in a test solution of 0.5 M sulfuric acid solution containing Fc^+^ at scan rate of 100 mV·s^−1^. The stabilized voltammograms were obtained after 10 cycles between the upper and lower potential limits of the cyclic voltammogram. In the scan for positive potentials, we observed an oxidation peak at 0.53 V that corresponded to the oxidation peak of Fc to Fc^+^. In the reverse scan, we observed a reduction peak at 0.40 V that corresponded to the reduction of Fc^+^ to Fc. The peak potential separation between anodic and cathodic features (Δ*E_p_*) was 130 mV. A fast, reversible, one-electron transfer would ideally have a Δ*E_p_* = 59 mV at 298 K. The discrepancy from this ideal value was mainly attributed to slow electron transfers. Figure 1 also shows the stabilized cyclic voltammogram of a GC electrode modified with SWCNT@SiO_2_ prepared in equivalent conditions to the previous electrode. The shape of the voltammogram was similar to the previous one, but the peak potential separation between anodic and cathodic features was 120 mV. This indicated that the response of the redox probe was more reversible in the present case than in SiO_2_-modified electrodes in the absence of carbon nanotubes. It also indicated that these species can improve the electron transfer after their incorporation into the silica matrix.

**Figure 2:**
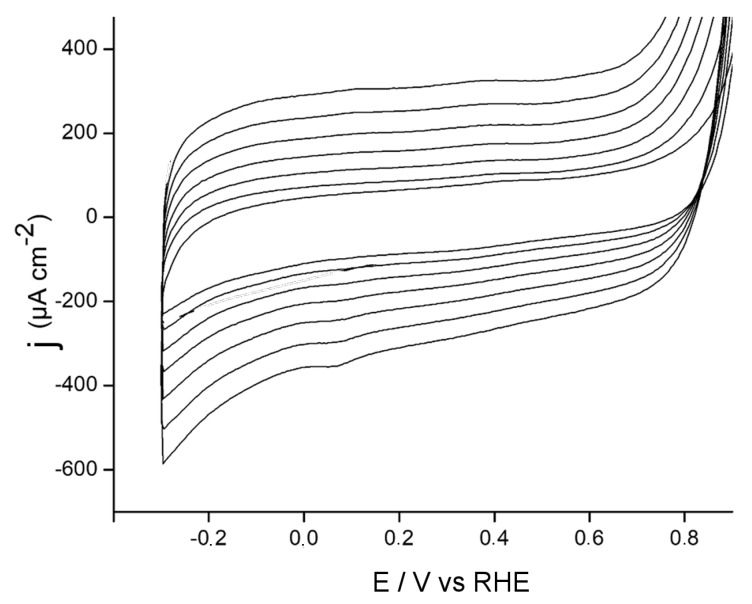
Cyclic voltammetric scans of an SWCNT@SiO_2_ electrode in a solution of 3,4-ethylendioxythiophene (EDOT) in poly (sodium 4-styrenesulfonate) (PSS). Anodic limit of 1.0 V. Scan rate of 100 mV s^−1^.

Since SWCNT may remain electrically isolated within the dielectric silica matrix, a good strategy to improve the performance of this electrode is the growth of a conducting polymer through the silica functionalized electrodes. Figure 2 shows the electrochemical synthesis of PEDOT-PSS through a SWCNT@SiO_2_-modified electrode.

The first potential cycle was a featureless voltammetric profile and was recorded until a potential value above 0.80 V was reached. This point corresponded to the onset potential of EDOT monomer oxidation and, consequently, to the formation of PEDOT-PSS. The inversion potential was set at 1.0 V to obtain a suitable growth rate of the polymeric material. On subsequent potential scans, the presence of a current plateau in the potential region between −0.2 and 0.8 V, showing capacitive features and an increasing voltammetric charge, was observed. This feature was assigned to the growth of PEDOT-PSS across the silica matrix. 

**Figure 3:**
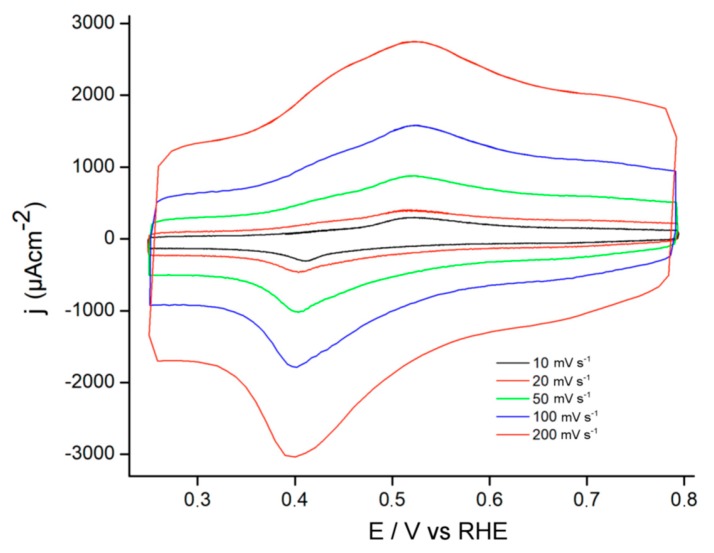
Stabilized cyclic voltammograms of an SWCNT@SiO_2_-PEDOT-PSS electrode in a solution of 1.0 mM FcFP_6_ in 0.5M H_2_SO_4_ at different scan rates.

Following the synthesis process, the electrodes coated with the polymeric films were rinsed with water and then immersed in a solution containing Fc^+^ with 0.5 M acid sulfuric solution. The voltammetric response of PEDOT-PSS electrosynthesized on the SWCNT@SiO_2_ electrode is shown in Figure 3. 

The voltammetric responses of SWCNT@SiO_2_-PEDOT-PSS presented clear current coming from capacitive processes of the conducting polymer at potentials lower than 0.4 V. The Fc/Fc^+^ redox processes appeared at around 0.5 and 0.4 V for oxidation and reduction process, respectively, at the different scan rates.

**Figure 4:**
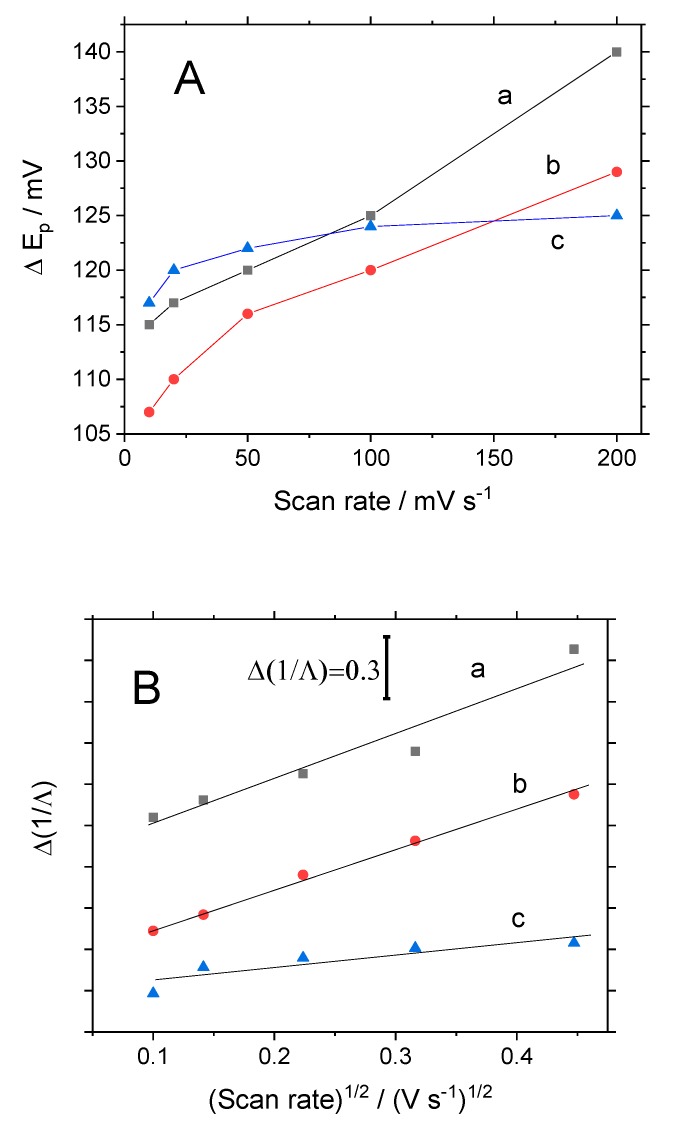
(**A**) Voltammetric peak potential separation between oxidation and reduction process of ferrocene as a function of the voltammetric scan rate for different electrodes. (**B**) Variation of the reciprocal of the Matsuda–Ayabe Λ parameter as a function of the square root of the voltammetric scan rate for different glassy carbon (GC)-modified electrodes. (**a**) SiO_2_; (**b**) SWCNT@SiO_2_; (**c**) SWCNT@SiO_2_-PEDOT-PSS.

To go into detail about the behavior of the modified electrodes, a kinetic analysis of their electrochemical performance was carried out. The kinetic reversibility of an electrochemical reaction can be evaluated from cyclic voltammetry experiments due to the Nicholson method, by making use of the values of peak potential separation at different scan rates. Figure 4A presents these values for the different electrodes immersed in the test solution of Fc^+^.

As observed for SiO_2_-modified electrodes at a low scan rate (10 mV·s^−1^), the value of peak potential separation was 115 mV but when the scan rate was increased, this parameter sharply increased reaching values of around 140 mV at 200 mV·s^−1^. A similar trend was observed for the SWCNT@SiO_2_ electrode, although the peak potential separation was lowered by the presence of the nanotubes inserted in the matrix. This indicated that a higher scan rate drove to a lower reversibility. The behavior of the SWCNT@SiO_2_-PEDOT-PSS electrode is completely different, and we can observe a lower dependency of the reversibility with the scan rate.

From the peak potential separation, we can obtain the Λ parameter defined by Matsuda and Ayabe [28]. It is usually assumed that an electrode process will be kinetically reversible for Λ > 15, quasireversible for 15 ≥ Λ ≥ 0.001 and irreversible for Λ values lower than 0.001. In the present case, the silica-modified electrode presents values of Λ ranging from 0.69 (at 10 mV·s^−1^) to 0.45 (at 200 mV·s^−1^). The SWCNT@SiO_2_ electrode presents Λ from 0.85 to 0.54 and the composite SWCNT@SiO_2_-PEDOT-PSS have Λ values from 0.67 to 0.57. In all cases, this probe can be categorized as quasireversible for these electrodes. The relationship between the standard rate constant, *k*^0^, for the electron transfer of the electrochemical reaction and the Λ parameter, was shown by Matsuda [28]:

(1) $$\frac{1}{\Lambda }=\frac{1}{k^{0}}{\left(\frac{nFD}{RT}\right)}^{1/2}\upsilon^{1/2}$$

A representation of the reciprocal of Λ against the square root of the scan rate allows the determination of the heterogeneous rate constant from the slope of each curve for each material. Figure 4B shows this plot where a linear trend is observed for all the electrodes. From the slope, the values of *k*^0^ were determined. The electrode modified with silica presented a value of 1.61 × 10^−2^ cm·s^−1^, whereas the electrode with carbon nanotubes (SWCNT@SiO_2_) presented a slightly higher electron transfer of 1.74 × 10^−2^ cm·s^−1^. Finally, the greatest result was the composite electrode where the rate transfer was enhanced to a value of 5.47 × 10^−2^ cm·s^−1^, indicating that the nanotubes were properly connected to the electrode support.

The electroactive area for electron transfer can be also determined from the voltammetric measurement. In this case, the classical Randles–Sevcik equation can be only applied to reversible systems:

(2) $$j_{p}\left(rev\right)=2.687\times 10^{5}ACn^{3/2}{\left(D\upsilon \right)}^{1/2}$$

where *j_p_(rev)* is the current density for a reversible redox process (this current density is referred to the geometric area of the electrode), A is the real electroactive area for the electron transfer, (this is a unitless parameter also called the roughness factor), *C* is the concentration of the redox probe (in mol cm^-3^), *n* is the number of electrons transferred, *D* is the diffusion coefficient (cm^2^·s^−1^) of the redox probes and *υ* is the scan rate (V·s^−1^). The application of this equation to both quasireversible and irreversible systems is only possible after the correction of the experimental peak current (*I_p_*):

(3) $$j\left(rev\right)=\frac{j_{p}}{k\left(\Lambda \right)}$$

where *k*(Λ) is an adimensional parameter defined by Matsuda and Ayabe, which accounts for the kinetic factor governing the peak current.

**Figure 5:**
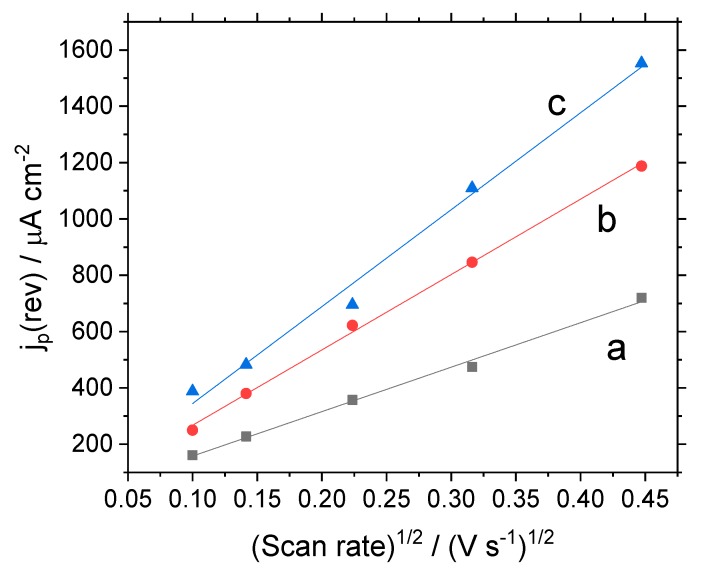
Randles–Sevcik plot for ferrocene oxidation with different GC-modified electrodes: (**a**) SiO_2_; (**b**) SWCNT@SiO_2_; (**c**)SWCNT@SiO_2_-PEDOT-PSS.

Figure 5 presents the Randles–Sevcik plots of peak current vs. the square root of the scan rate. For the SiO_2_-modified electrode, the Fc^+^/Fc reaction occurs at an effective electrode area of A = 1.10, which is the real area that is very similar to the geometric area of the glassy carbon support. Upon the introduction of the electrocatalytic carbon nanotubes, the value of A reached 1.86, indicating that some nanotubes were directly connected to the electrode support. Finally, the SWCNT@SiO_2_-PEDOT-PSS composite electrode presented a value of A = 2.39, which is indicative of a proper connection of some remaining nanotubes dispersed in the silica with the GC support.

### 3.2. Surface Characterizations by Scanning Electron Microscopy

**Figure 6:**
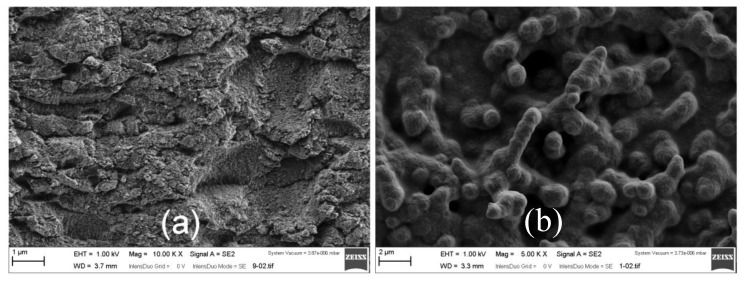
Scanning electron microscopy (SEM) images of GC-modified electrodes: (**a**) SWCNT@SiO_2_; (**b**)SWCNT@SiO_2_-PEDOT-PSS.

The scanning electron micrographs of the SWCNT@SiO_2_ electrode and modified electrode with PEDOT-PSS are shown in Figure 6. 

For the SWCNT@SiO_2_ electrode, the electrochemically deposited layer looks homogeneous all over the surface, with randomly distributed pores showing an approximate diameter of around 2 µm. These results revealed the granular morphology and that the edges and angles of SWCNT became smooth and PEDOT-PSS was deposited onto the surfaces of the electrode. On the other hand, this resulting SWCNT@SiO_2_-PEDOT-PSS electrode shows a rounded edge and broad (near one micron) dendritic structures, which provide the modified sample with an aspect quite different from the smoother, unmodified polymer films shown in Figure 6b. These striking architectures were formed by PEDOT-PSS emerging from the silica material and their shape is a consequence of the patterned growth of the PEDOT forced by the structure of silica. 

## 4. Conclusions

The electrochemical behavior of the composite electrodes was tested against the ferrocene redox probe. The SWCNT@SiO_2_ electrodes contain electrocatalytic nanotubes dispersed within its structure, as demonstrated by the improvement of the electrochemical performance in terms of heterogeneous rate constant and the electroactive area. However, the modest improvement of those parameters indicated that a major part of the SWCNT remains electrically isolated from the electrode support. PEDOT-PSS films were synthesized successfully by reactive electrochemical polymerization across SWCNT@SiO_2_-modified electrodes. The SWCNT@SiO_2_-PEDOT-PSS composite electrodes obtained a heterogeneous rate constant more than three times higher than the electrode without conducting polymer. Similarly, the electroactive area was also enhanced to almost double of the supporting GC electrode for the SWCNT@SiO_2_-modified electrodes. A further increase of electroactive area was observed for the SWCNT@SiO_2_-PEDOT-PSS composite electrodes.
